# Sheep and wheat domestication in southwest Asia: a meta-trajectory of intensification and loss

**DOI:** 10.1093/af/vfab010

**Published:** 2021-06-19

**Authors:** Daniel Fuks, Nimrod Marom

**Affiliations:** 1 McDonald Institute for Archaeological Research, Department of Archaeology, University of Cambridge, Downing St, Cambridge, UK; 2 Martin (Szusz) Department of Land of Israel Studies and Archaeology, Bar-Ilan University, Ramat GanIsrael; 3 Department of Maritime Civilizations and the Leon Recanati Institute for Maritime Studies, University of Haifa, Haifa, Israel

**Keywords:** agricultural diversity, globalization, Neolithic package, origins of agriculture, pastoralism

**Implications**

- Biologists since Darwin considered domestication a model for the study of evolution; we argue that domestication may also be a model for the study of globalization.

- The long-term history of wheat and sheep domestication exemplifies the intensification of relationships between humans and a small number of species native to southwest Asia, which includes long-term globalizing processes.
- Specific indicators are offered for tracking the long-term globalization of sheep and wheat, with reference to production intensity, geographic diffusion, and diversity.

## Introduction

Domestication as here understood is one outcome of human–environment interactions whereby certain plants and animals undergo genetic changes resulting from their close relationship with humans, including increasing reliance on humans for survival and reproductive success. Domestication is thus an ongoing process and may be viewed as part of an even broader process of intensification in the relationships between humans and certain plants and animals, including hunting/gathering, herding/cultivating, specialized agriculture/pastoralism, and, recently, genetic engineering. It should be emphasized that these are not stages in a necessarily directional process, but these categories do represent a scale of intensification, at least in the strict agricultural sense of more plant/animal product per unit land (Harris, 1989). Domestication has enhanced evolutionary fitness for domesticated species, humans included (Rindos, 1984). It is thus a type of symbiosis, the study of which contributes to broader understandings of evolution (Ladizinsky, 1998; Larson et al., 2014). In the case of wheat and sheep, symbiotic relationships developed not only between sheep–humans and wheat–humans but also between wheat–sheep, especially as a result of intensified management strategies, for example, grazing on stubble in harvested fields, foddering and manuring, and forest clearing. As has long been appreciated, these relationships involve biological and cultural aspects (e.g., Rindos, 1984; Ingold, 1996).

Whereas the tradition of studying domestication as a model for evolution goes back to Darwin, we argue that domestication research also offers a model for the study of globalization. This suggestion ensues from the insight that several components of the meta-trajectory outlined below as intensifying relationships between humans, wheat, and sheep, are manifest in many other ongoing economic, social, and ecological processes. These can be broadly summarized as “globalization” in the widely accepted sense of intensifying worldwide interconnectedness, including in economic, cultural, political, and environmental spheres (Held et al., 1999: 2). Our long-term history of sheep and wheat domestication agrees with the consensus view that contemporary globalization represents new levels of intensification, but also that it has much earlier roots than is commonly acknowledged. Finally, we offer specific indicators for tracking the long-term globalization of sheep and wheat domestication, with reference to production intensity, geographic diffusion, and diversity.

## Sheep

**Figure 1.**
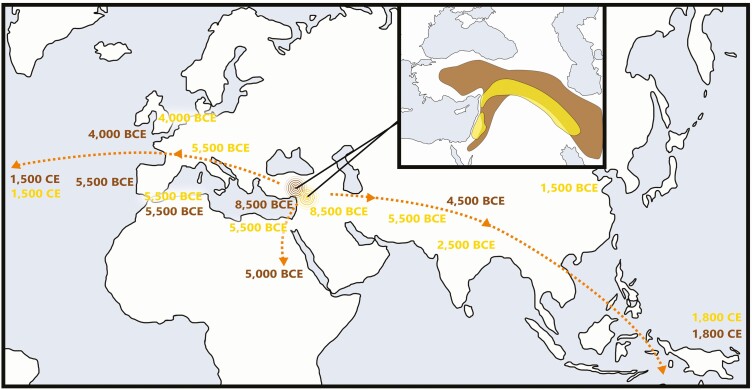
Long-term diffusion of domesticated sheep and wheat from their centers of origin. Schematic portrayal of the spread of domesticated sheep (brown) and wheat (yellow) across Eurasia and beyond, with approximate dates of arrival in key areas. Insert shows approximate phytogeographic distribution of wild progenitors, wild mouflon sheep (*Ovis orientalis*) in brown and wild emmer wheat (*T. turgidum* subsp. *dicoccoides*) in yellow.

Sheep are the second most abundant ruminant livestock animal after cattle (Gilbert et al., 2018) and have been bred intensively to optimize wool, milk, fat, or meat production. In southwest Asia, sheep were among the first domesticated livestock. Together with goats, cattle, and swine, they make up the key animal components of the Neolithic “package,” which subsequently spread throughout the globe (Figure 1). Sheep were domesticated from the mouflon (*Ovis orientalis*), with little evidence for genetic input of other wild congenerics (*O. vignei*, *O. nivalis*, *O. ammon*) to extant or archaeological populations (Deng et al., 2020). Domesticated sheep have descended from several mouflon lineages, suggesting a complex population history (Pedrosa et al., 2005).

The date of first appearance of sheep in Cyprus at ca. 8000 BCE (Vigne et al., 2011) is a solid *terminus ante quem* for management, as sheep are not part of the native Pleistocene fauna of this island and so must have been introduced there. It is more difficult to pinpoint the somewhat earlier intensification within the Pleistocene range of mouflon in southwest Asia. Early evidence for domestication is found in the reduction of caprine body size in sites from the upper Euphrates basin (Nevalı Çori) in the mid-9th millennium BCE (Peters et al., 2005). A broadly similar date has been obtained from Aşıklı Höyük in Anatolia (Stiner et al., 2014). From this cradle of domestication in southwest Asia, sheep spread across Anatolia (Arbuckle, 2008), to the southern Levant in the 8th millennium BCE (Horwitz et al., 1999), to Crete by 7000 BCE (Jarman and Jarman, 1968), to the Greek mainland by 6500 BCE (Davis and Simões, 2020), and to the Iberian peninsula and the Maghreb by ca. 5500 BCE (Kandoussi et al., 2020; Figure 1). By 4000 BCE sheep were present in northern Europe (Rowley-Conwy, 2013). The earlier 5th millennium BCE also witnessed the first appearance of domesticated sheep in China (Dodson et al., 2014). Sheep and other domestic livestock first appear in Africa by 5000 BCE (Muigai and Hanotte, 2013), reaching the inner, southern, and western parts of the continent appreciably later, in the 1st and 2nd millennia BCE (Marshall and Hildebrand, 2002).

The transition from hunting to domestication of sheep has tracked multiple paths during the southwest Asian Neolithic (Makarewicz, 2013). Different combinations of herding, hunting, and farming were tried—not all of them successful or sustainable—as revealed by the archaeological record. For example, nondiscriminant early slaughter of animals from both sexes, against modern utilitarian logic, appears in Aşıklı Höyük (Stiner et al., 2014); slaughter of younger males seems to have become a widespread management tactic only by the end of the 8th millennium BCE (Arbuckle and Atici, 2013). Foddering has been suggested in Neolithic southern Jordan (Makarewicz and Tuross, 2012) and Anatolia (Miller and Marston, 2012), while manipulation of lambing season has been identified in Neolithic France, 5th millennium BCE (Tornero et al., 2020). Mosaics of agricultural and transhumant practices are found across southwest Asia (Martin, 1999; Arbuckle and Hammer, 2019). The first evidence for vertical transhumance between mountains and plains appears in 6th millennium BCE Anatolia (Makarewicz et al., 2017).

Another element of pastoral complexity concerns choices regarding which domestic species to raise and in what proportions, giving rise to an endless variety of possibilities evident in the diversity of pastoralists’ herding strategies. For instance, a manifold range of considerations determines the logic behind the ratio between the two caprine species in traditional southwest Asian herding strategies (Redding, 1981; Cribb, 1984). In general, sheep products (meat, milk, wool) are considered more valuable than those of goats in southwest Asia, but sheep require more water, more herbaceous pasture, and therefore larger ranges. Goats have fewer dietary and water requirements, breed faster, and are more suitable as livestock for the risk-averse or when human and land resources are limited. The complexity of early domestication processes is echoed in the multiple pathways through which livestock, among them sheep, were integrated into subsistence practices in different regions of the world. Whereas in Europe they were part and parcel of the agricultural package that spread westward and northward from southwest Asia, in Africa a slower process of assimilation appears to have been the rule (Zeder, 2017).

The utilization of secondary products such as milk and wool (Sherratt, 1983) has been an important consideration for keeping sheep throughout history. There is evidence for the use of sheep’s milk already in Neolithic diets (Hendy et al., 2018), as another strategy for obtaining dietary value. Wool, however, is a newer currency through which long-range social debts can be committed: it represents an ability to harness marginal lands for the production of tradeable goods, which support a complex economic structure (McCorriston, 1997). The development of specialized breeds for wool production is suggested to have occurred for the first time in late 4th millennium BCE Mesopotamia (Algaze, 2009), based on iconographic representations of coil-horned rams with fleeces, which replaced a large corkscrew-horned breed. Significantly, this change can be traced in the biometry of sheep in the region (Vila and Helmer, 2014). Large-scale wool production, alongside other types of specialized herding (e.g., fat-tailed sheep for food and sacrifice), is associated with the late 3rd millennium BCE Ur III state (Stepien, 1996) and is known in southwest Asia and the eastern Mediterranean throughout the 2nd–1st millennia BCE (Killen, 1964). Specialized breeding further intensified under subsequent empires, such as those of the Assyrian, Classical, and Islamic worlds (Davis, 2008; Marom and Herrmann, 2014).

Recent centuries have seen a revolution in the domestication relations between sheep and humans. The *mesta* system of Merino shepherding in medieval Spain and the British wool industry associated with the bursting international trade of the industrial revolution, exemplify intensification in the context of early capitalistic growth (Klein, 1920). In modern times, this process continues in the modern Australian Merino fiber industry, exemplifying new levels of agropastoral intensification in the historical process of globalization. Here a former British colony utilizes a North African breed to supply diverse markets worldwide, including that of Olympic sports. Scientific advances in selective breeding over the last 200 years, and its increasing efficiency due to artificial insemination within the context of factory farming, has caused a sharp decline in genetic diversity relative to population size (estimated at ~1.2 billion sheep worldwide). The effective population size of many breeds is now below 50, local breeds have disappeared, and the cultural diversity associated with pastoral production is dwindling. Following the genetic cloning of Dolly in 1997, the conceptual path to intrusive genetic intervention in sheep breeding was laid. Today, CRISPR/Cas9 edited Australian Merino sheep that can produce both fine wool and quality meat represent the materialization of this path (Crispo et al., 2015), topping an already mounting concern for the genetic future of sheep due to diversity loss (Taberlet et al., 2011).

## Wheat

Wheat is the most important source of food grain for humans today and the largest primary commodity (FAO, 2014). Although wild wheats are native only to southwest Asia, domesticated wheat has spread throughout the globe (Figure 1). Today, wheat fields occupy more land than any other crop on the planet (FAO, 2014, 2020), representing an extreme case of domestication and diffusion. “Wheat” refers to a genus of grasses (*Triticum*). A natural classification system groups wheat species by chromosomal ploidy (multiples of distinct sets of chromosomes) and combinations of distinct genomes (Table 1; van Slageren, 1994; Zohary et al., 2012: 23–9; Haas et al. 2019). Wheat subspecies are further differentiated as wild/domesticated and hulled/free-threshing and by number of kernels per spikelet—genetic traits that have clear phenotypic expressions in wheat spikelet morphology (Hillman et al., 1996).

**Table 1.** Natural classification of wheat species (after Zohary et al., 2012, Table 3)

Ploidy	Genomes	Species name	Wild/domestic forms
Diploid (2n)	AA	*Triticum monococcum* L.	Wild & domestic
Diploid (2n)	AA	*T. urartu* Tuman	Wild
Tetraploid (4n)	BBAA	*T. turgidum* L.	Wild & domestic
Tetraploid (4n)	GGAA	*T. timopheevii* Zhuk.	Wild & domestic
Hexaploid (6n)	BBAADD	*T. aestivum* L.	Domestic
Hexaploid (6n)	GGAAAA	*T. zhukovskyi* Men. & Er.	Domestic

**Figure 2.**
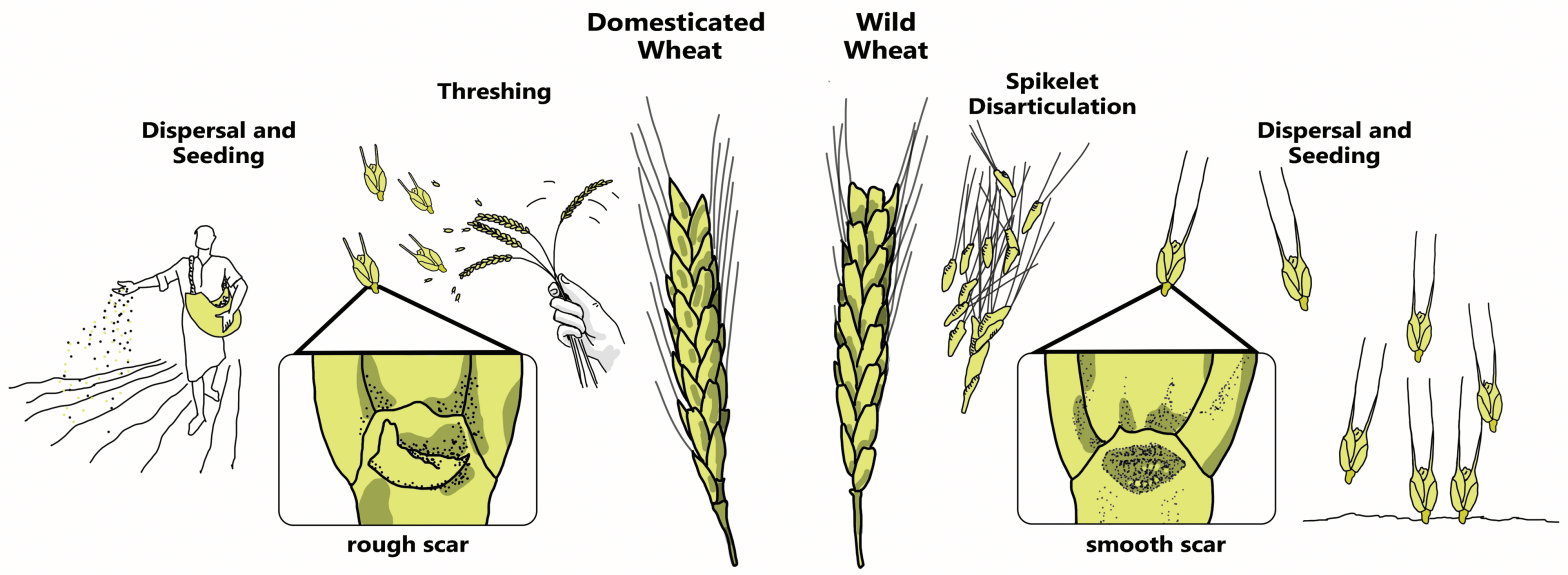
Domesticated vs. wild wheat. The primary distinction between wild and domesticated wheats is based on spikelet morphology. In wild wheats, spikelets act as dispersal units, disarticulating upon maturity, and leaving a smooth scar on the rachis segment (right). In domesticated wheats, spikelets are released only upon threshing; detachment of spikelets from the spike leaves a rough scar on the rachis fragment (left). Unlike wild wheats, domesticated wheats rely on humans for dispersal and seeding.

The key trait distinguishing wild and domesticated cereals is spikelet brittleness. In wild cereals, the spikelet acts as a dispersal unit, disarticulating from the ear at maturity, dispersing by different vectors, and implanting itself in the ground with the aid of its awns (Figure 2). Spontaneous disarticulation upon maturity—which leaves a smooth scar on the rachis segment—makes it difficult to harvest fully ripe wild cereals from the ear, although a small percentage (ca. 10%) of nonbrittle spikelets are retained at the base of wild cereal ears (Kislev, 1989). Prior to domestication, Epipaleolithic people, ca. 21–9.7 ka Cal BCE, gathered wild wheat, among other grasses (Weiss et al., 2004; Arranz-Otaegui et al., 2018a), for grinding and food preparation (Nadel et al., 2012; Arranz-Otaegui et al., 2018b), and may have even engaged in cultivation of wild cereals (Snir et al., 2015). Growing archeobotanical evidence suggests predomestication cultivation of wheat and other grasses in the PPNA, 9.7–8.8 ka Cal BCE (Weiss et al., 2006; cf. Abbo et al., 2021).

In domesticated cereals, the spikelet no longer acts as a dispersal unit and does not disarticulate upon ripening. For dispersal and germination, domesticated wheat relies on planting by humans. The tough rachis segments may separate by tearing at the internodes, leaving rough scars (Figure 2). Increasing proportions of rachis segments with rough scars in archeobotanical assemblages place initial domestication of emmer and einkorn wheat in the EPPNB, 8.8–8.3 ka Cal BCE, proliferating in the MPPNB, 8.3–7.7 ka Cal BCE, throughout southwest Asia (Zohary et al., 2012: 36–38, 41–43; Arranz-Otaegui et al., 2018a). However, archeobotanical data suggest that it took some 2000 yr between initial domestication as represented by >20% nonbrittle rachises and full morphological domestication of >80% domestic rachises (Fuller et al., 2018; cf. Abbo et al., 2021). Over the same period, increased kernel breadth is an additional marker of domestication (Fuller et al., 2018).

**Figure 3.**
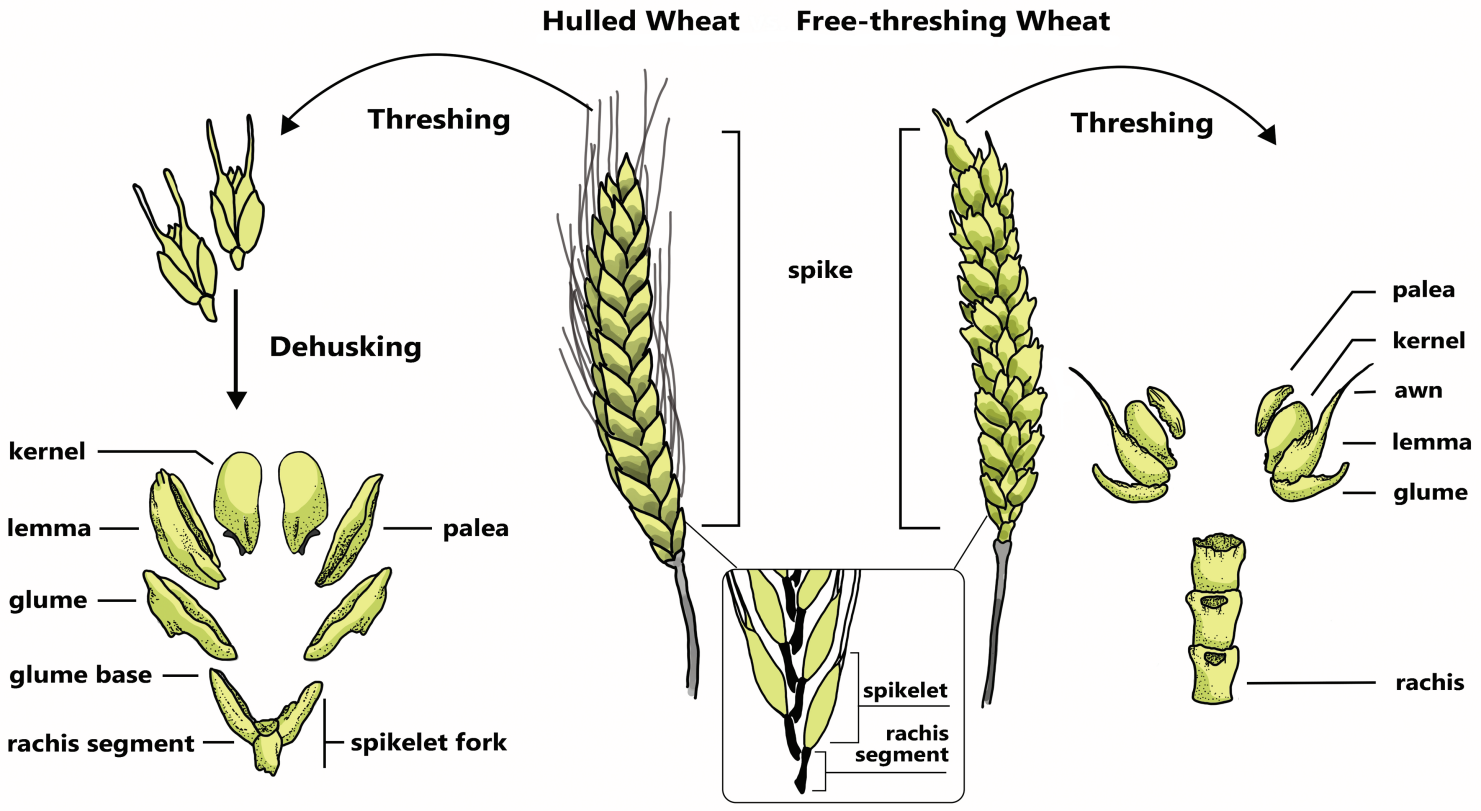
Hulled vs. free-thresing wheat. Domesticated wheats are either hulled or free-threshing. In hulled wheats (left), kernels are tightly enclosed in their glumes such that threshing results in intact spikelets. To release the kernels, they must be dehusked. Of the resultant chaff, spikelet forks are a tell-tale identifier of hulled wheats, commonly found in archeobotanical assemblages. In free-threshing wheats, threshing alone is sufficient to release kernels and chaff, which includes rachises indicative of free-threshing wheats.

The first domesticated wheats were, like their wild progenitors, “hulled” or “glume” wheats, meaning that their kernels are tightly enclosed in the spikelet by tough glumes that do not break off during threshing and which therefore require dehusking to release the kernels (Figure 3). In addition to einkorn and emmer, an apparently distinct domestication of Timopheev’s wheat (Table 2) is indicated by a recent archeogenetic study identifying as such the extinct “new glume wheat” known from the Neolithic archeobotanical record in Anatolia and the Balkans (Czajkowska et al., 2020). New glume wheat was cultivated for millennia before its extinction, but other forms of domesticated Timopheev’s wheat are extant (Jones et al., 2000).

**Table 2.** Some important wheat subspecies (after van Slageren 1994)

Subspecies	Wild/domesticated	Hulled/naked	Common name
*T. monococcum* L. subsp. *aegilopoides* (Link) Thell.	Wild	Hulled	Wild einkorn
*T. monococcum* L. subsp. *monococcum*	Domesticated	Hulled	Domesticated einkorn
*T. turgidum* L. subsp. *dicoccoides* (Asch. & Graebn) Thell.	Wild	Hulled	Wild emmer
*T. turgidum* L. subsp. *dicoccum* (Schrank) Thell.	Domesticated	Hulled	Domesticated emmer
*T. turgidum* L. subsp. *durum* (Schrank) Thell.	Domesticated	Naked	Durum, aka macaroni/hard wheat
*T. timopheevii* Zhuk. subsp. *armeniacum* (Jakubz.) van Slageren	Wild	Hulled	Wild Timopheev’s wheat
*T. timopheevii* Zhuk. subsp. *timopheevii*	Domesticated	Hulled	Domesticated Timopheev’s wheat
*T. aestivum* L. subsp. *spelta* (L.) Thell.	Domesticated	Hulled	Spelt
*T. aestivum* L. subsp. *aestivum*	Domesticated	Naked	Bread wheat
*T. zhukovskyi* Men. & Er.	Domesticated	Hulled	Zhukovsky’s wheat

“Free-threshing” or “naked” wheat kernels are surrounded by thinner glumes, which release the kernels upon threshing, as in tetraploid durum wheat and hexaploid bread wheat (Figure 3). Free threshing makes postharvest processing more efficient and was selected for relatively rapidly following initial domestication, as is evident from the Middle/Late PPNB (Hillman et al., 1996; Zohary et al. 2012: p. 24). Kislev described an early and now extinct form of free-threshing tetraploid wheat, *T. turgidum* subsp*. parvicoccum* Kislev, which may have been an intermediary subspecies in the evolution of durum from emmer (Kislev, 1979, 2009; cf. Nesbitt, 2001). Free-threshing wheats replace hulled wheats in Early Bronze Age (ca. 3300–2100 BCE) Anatolia and northern Syria; the same occurred in Late Bronze Age (ca. 1550–1200 BCE) Canaan, although hulled wheats did not phase out entirely and they continued to dominate in the Aegean into the Iron Age (ca. 1200–600 BCE) (Riehl and Nesbitt, 2003; Frumin et al., 2019). Despite their greater processing costs and generally lower gluten content, hulled wheats’ persistence is probably due to their greater resistance to poor soil conditions, fungal diseases, and insect pests (Nesbitt and Samuel, 1996).

Another major milestone in wheat domestication is the advent of hexaploid wheat by the 7th millennium BCE (Bogaard, 2016), from spontaneous hybridization of tetraploid domesticated emmer with the diploid wild grass, *Aegilops tauschii* Coss. (Zohary et al., 2012: 33, 47). The latter contributed the D genome, conferring greater adaptability to non-Mediterranean climates (Zohary et al., 2012: 49). Although not exclusive to hexaploid wheats, the evolution of spring wheat, especially via flowering time adaptability to diverse temperatures, soil moisture, and day length (Kamran et al., 2014), further contributed to their widespread diffusion. Free-threshing hexaploid wheat formed a part of Neolithic farming in Europe by the 3rd millennium BCE (Nesbitt, 2001), while also spreading eastward to India by 2500 BCE, and central China by 2000 BCE, as well as wider latitudes and higher altitudes of Eurasia (Liu et al., 2017). Hulled hexaploid wheats, like spelt, became important to many local economies in Europe from the Bronze Age to premodern times (Nesbitt, 2001, 2005).

By the end of the southwest Asian Neolithic, all the major wheat types described above were under cultivation in Eurasia, with wide inter-regional diversity (Fuller et al., 2018). Domesticated emmer wheat (along with barley) became a staple of the Early Bronze Age Levantine city-states (e.g., Hopf, 1983), although its cultivation in some early agricultural settlements of the period was unsustainable and unsuccessful (White et al., 2014). Among later empires, in 7th c. BCE Assyrian Israel a regional production strategy apparently involved wheat grown in Judea to feed residents of Ashkelon, freeing land closer to the ports for Mediterranean-export viticulture (Faust and Weiss, 2005). The globalizing Hellenistic-Roman economies apparently effected a transition from hulled emmer to free-threshing durum wheat in their Egyptian breadbasket during the first few centuries CE (Cappers, 2016).

Hulled wheats (at all ploidy levels) gradually phased out of cultivation for their lesser value to commercialized and globalized economies of antiquity and modern times, particularly in tandem with 20th-century globalization of free-threshing hexaploid bread wheat cultivation (Nesbitt and Samuel, 1996). Hulled wheats survived under cultivation in mountainous pockets of western Eurasia, making a minor comeback as popular health foods in recent decades (Nesbitt and Samuel, 1996; Nesbitt, 2005). Today, tetraploid free-threshing durum, or “macaroni wheat,” accounts for some 5% global wheat production—much of which is grown in the Mediterranean basin (Royo et al., 2017). Hexaploid free-threshing “bread wheat” accounts for almost 95% of global production and is cultivated in nearly every country worldwide. Aside from enhanced adaptability, hexaploid free-threshing wheat’s commercial dominance is due to higher gluten content, making it the ideal bread wheat. Both bread wheat and durum are subject to the full efforts of modern crop improvement, including genetic engineering.

## Discussion

A powerful combination of southwest Asian plant and animal domesticates emerged in the Neolithic—an “agricultural package”—of which wheat and sheep are exemplary. Increasing evidence suggests that even after initial domestication, cultivation and livestock rearing developed by numerous and diverse pathways, including much trial and error (White et al., 2014; Honeychurch and Makarewicz, 2016). Although agriculture and pastoralism involve a significant focus on select few species compared with the many dozens utilized by hunter-gatherers, the success of southwest Asian food production may nonetheless be attributed to different forms of diversity inherent in the Neolithic package.

The most basic form of such diversity is that deriving from the combination of plants and animals. This not only provides a source of dietary diversity, as does hunting and gathering, but also an added level of risk management associated with agropastoral storage. Whereas wheat grains, among other cereals and legumes, can be stored in permanent settlements for food and sowing, sheep and other livestock are a highly mobile source of food and capital. Together, the combination of stationary and mobile storage provides a wide range of adaptations to environmental anomalies mediated by diverse cultural modes. The development of specialized nomadic pastoralism is a kind of intersociety adaptation on this theme, developed to maximize landscape exploitation by focusing grazing on regions less suitable for agriculture. This perspective is supported by the high degrees of interdependence between specialized pastoralists and farmers, alongside tensions over scarce land and sociocultural differences. Much of later southwest Asian history can be written in terms of these relationships and differences, following the lead of Ibn Khaldun (1958 [1377]). However, it is important to emphasize that rather than a simple binary nomadic pastoralist/sedentary farmer dichotomy, these categories represent continuous spectra with potentially infinite combinations and interrelations.

A different type of diversity contributing to agropastoral buffering capacity involves the set of trade-offs between sheep and wheat vis-à-vis their respective counterparts, goats and barley. Both sheep and goats provide meat, milk, and hides; both wheat and barley provide kernels for food and fodder, as well as chaff and straw for fodder, kindling, building, and other crafts. However, while offering essentially the same products, each member of the pair has slightly different ecological needs and adaptive qualities, with barley and goats generally representing the hardier counterparts to the higher-valued products of sheep and wheat among most ancient and modern southwest Asian cultures. These differences may be exploited in various ways and circumstances, including risk management. For example, drought-tolerant barley often succeeds where wheat crops fail, while slightly different ripening times between wheat and barley in southwest Asia offer a buffer against subseasonal precipitation anomalies.

In addition to interkingdom and intergenus diversity just discussed, intragenus and intraspecies diversity presents another gamut of possibilities for economic exploitation, utilized by breeders for millennia. For instance, changes under domestication to seasonal cyclicity in reproduction, involving flowering time adaptations for wheat and multiple lambing seasons in sheep, were key to their global diffusion. 

Just as genetic diversity has influenced the globalization of sheep and wheat, human socioeconomic globalization has affected their genetic diversity. The spread of these species to diverse and often remote regions catalyzed the development of numerous breeds and varieties (via selection for locally adapted traits, cultural preferences, genetic bottlenecking, etc.), creating a global force for increased intraspecies diversity—a diversity which most people throughout history were unaware of. Contemporary globalization has made this agriculturally significant diversity uniquely accessible in theory, as through gene banks, while causing declining diversity of cultivated/herded stock in practice as landraces become marginalized and extinct. These two countercurrents epitomize contemporary globalization generally: increased awareness of global diversity thanks to heightened connectivity between disparate regions on the one hand, and increased uniformity in cultural, social, and economic spheres on the other hand. If globalization widely conceived is a stage in the intensification of economic, cultural, political, and environmental interconnectedness, the globalization of sheep and wheat is a stage in domestication and agropastoral intensification, the tracking of which may broaden our understanding of contemporary globalization. We propose the following indicators for sheep and wheat intensification with relevance to long-term globalization:

(1) Sheep:goat and wheat:barley ratio
  Centralized and market-oriented production appear to favor both wheat and sheep vis-à-vis barley and goats, as well as specific varieties/breeds of each. By the Early Bronze age, wheat and sheep were involved in increasingly extractive, landscape-altering human lifeways, which was part and parcel of the rise of urbanism and empires. Whether in Ur III, the Assyrian Levant, or the Roman Mediterranean, local maxima in wheat and sheep production over time attest to heightened societal complexity, defined simply as increasing energetic inputs and problem-solving outputs (Tainter, 1990).
(2) Population density
  Increasing population density may occur on highly local and global levels. The former may involve, for example, intensive rearing of large herds in pens, supported by cultivated fodder. The latter includes global population levels of sheep and goats, which in a globalized world correlate with population densities in “core” areas.
(3) Geographical diffusion
  The extreme dispersal of wheat and sheep globally (Figure 1) has been used to explain modern Western global economic dominance (Diamond, 1997; cf. Frank and Gills, 1993). To chart this diffusion is to chart what may be the most basic precursor to globalization (Jones et al., 2011; Liu et al., 2019).
(4) Ratio of species population to number of extant agriculturally significant varieties and breeds.
  The globalization of wheat and sheep is also associated with increasing uniformity in the varieties and breeds being raised. In post-Neolithic times, this process includes gradual phasing out of einkorn and other hulled wheats, for example, and the global dominance of “bread wheat.”
(5) Geographic distribution of diversity in varieties and breeds.
  In a complex society as defined above, higher uniformity in varieties and breeds is expected along the major trade routes. Evenness in the geographic spread of rare landraces is expected to be a function of distance from primary economic and sociopolitical conduits.

**Figure 4.**
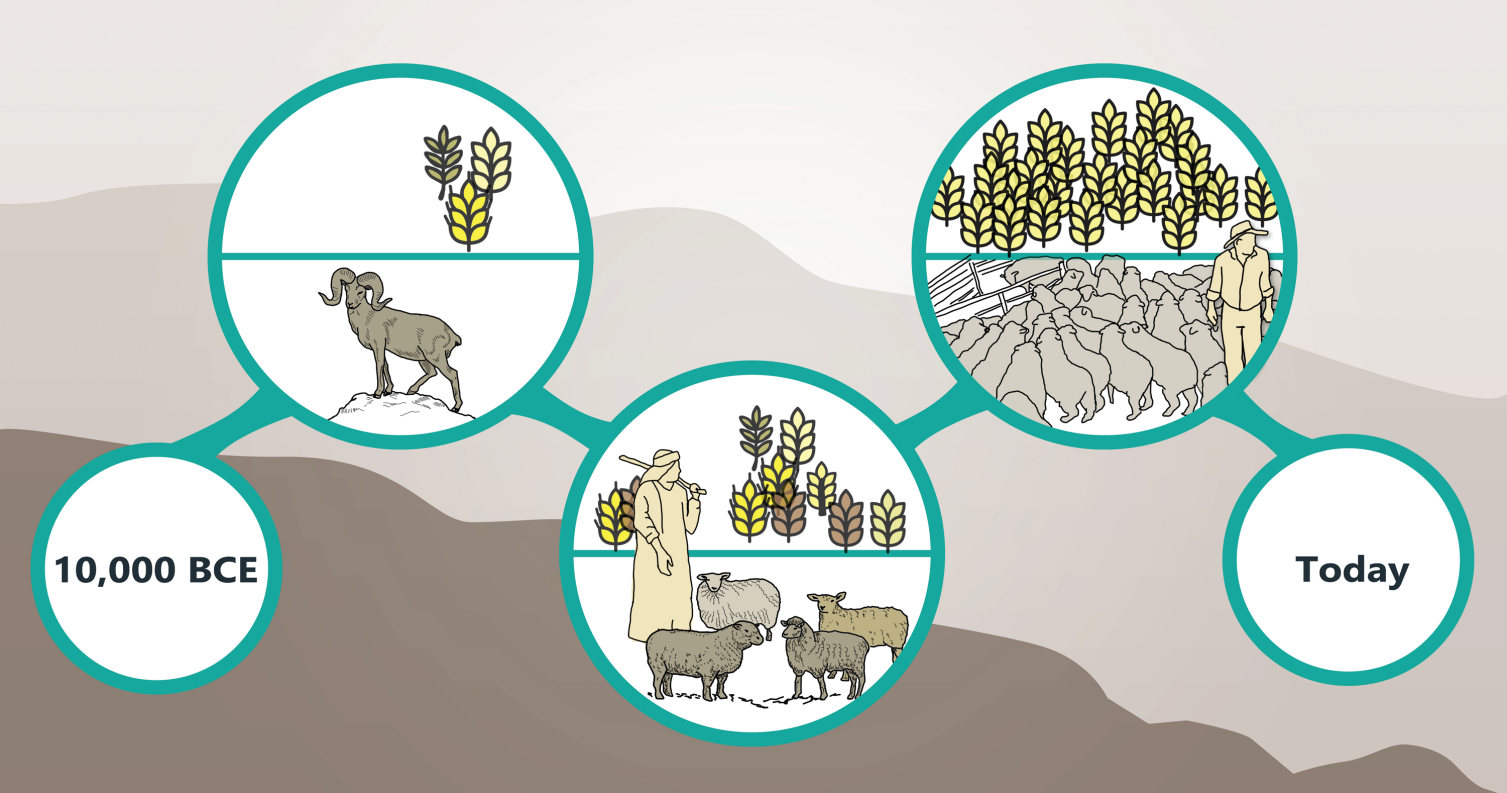
Population and diversity following domestication. Schematic portrayal of changes in domesticated sheep and wheat population and diversity over time.

Each of these indicators relates to three themes that are central also to contemporary globalization: production intensity, geographic diffusion, and diversity. More specifically, indicators (1) and (2) relate directly to production intensity; indicator (3) *is* geographic diffusion; while indicators (4) and (5) are agropastoral expressions of decreased cultural and genetic diversity (schematically portrayed in Figure 4). Thus, while many scholars view domestication as a model for evolution, domestication also offers a model for globalization. Sheep and wheat domestication exemplify globalization as a long-term historical phenomenon, which includes preference for output over risk aversion, increasing geographic diffusion and population density, as well as increasing awareness of global diversity and its relegation to collections of the past. We emphasize that these are neither continuous, directional, nor inevitable developments, and their integration in our synthesis of wheat and sheep domestication along a linear time progression should not be misunderstood as a ‘progress narrative’. The latter may be just as dangerous when applied to globalization as to evolution. The loss of biological and cultural diversity associated with agropastoral intensification spreads along the hyper-connected highways of globalization, as once the agricultural package comprising both taxa spread from southwest Asia across Eurasia through the ecological corridors afforded by the great river valleys. It may be that research into this meta-trajectory of intensification and loss, common to both sheep and wheat, may result in succoring through documentation a meager fraction of that loss for future generations.

## About the Authors

**Daniel Fuks** is an archeobotanist with academic interests in plant domestication and diffusion, ancient agriculture and pastoralism, ancient economic history, and Mediterranean history. He completed his MA and PhD at the Land of Israel Studies and Archaeology Department of Bar-Ilan University, Israel, and is now a British Academy Newton International Fellow at the McDonald Institute for Archaeological Research, University of Cambridge.

**Nimrod Marom** is an archeozoologist studying the interactions between animals, humans, and the environment in the Holocene and Late Pleistocene. He is a member of the Department for Maritime Civilizations at the University of Haifa, and PI of the DEADSEA_ECO project funded by the European Research Council.
